# Novel Allocation Strategies Can Boost Kidney Exchange Programs: A Monte Carlo Simulation

**DOI:** 10.3389/ti.2026.15423

**Published:** 2026-03-04

**Authors:** Mattheüs F. Klaassen, Marry de Klerk, Marije C. Baas, Hanneke Bouwsma, Laura B. Bungener, Maarten H. L. Christiaans, Twan Dollevoet, Kristiaan Glorie, Sebastiaan Heidt, Aline C. Hemke, Margriet F. C. de Jong, Judith A. Kal-van Gestel, Marcia M. L. Kho, Jeroen D. Langereis, Karlijn A. M. I. van der Pant, Claudia M. Ranzijn, Dave L. Roelen, Eric Spierings, Christina E. M. Voorter, Jacqueline van de Wetering, Arjan D. van Zuilen, Joke I. Roodnat, Annelies E. de Weerd

**Affiliations:** 1 Department of Internal Medicine, Erasmus MC Transplant Institute, University Medical Center Rotterdam, Rotterdam, Netherlands; 2 Department of Internal Medicine, Radboud University Medical Center, Nijmegen, Netherlands; 3 Department of Internal Medicine, Leiden University Medical Center, Leiden, Netherlands; 4 Department of Laboratory Medicine, University Medical Center Groningen, Groningen, Netherlands; 5 Department of Internal Medicine, Maastricht University Medical Center, Maastricht, Netherlands; 6 Erasmus School of Economics, Erasmus University Rotterdam, Rotterdam, Netherlands; 7 Erasmus Q-Intelligence, Erasmus University Rotterdam, Rotterdam, Netherlands; 8 Dutch Transplant Foundation, Leiden, Netherlands; 9 Department of Internal Medicine, University Medical Center Groningen, Groningen, Netherlands; 10 Department of Laboratory Medicine, Radboud University Medical Center, Nijmegen, Netherlands; 11 Department of Internal Medicine, Amsterdam University Medical Center, Amsterdam, Netherlands; 12 Department of Immunogenetics, Sanquin Diagnostic Services, Amsterdam, Netherlands; 13 Department of Immunology, Leiden University Medical Center, Leiden, Netherlands; 14 Central Diagnostics Laboratory and Center of Translational Immunology, University Medical Center Utrecht, Utrecht, Netherlands; 15 Department of Transplantation Immunology, Maastricht University Medical Center, Maastricht, Netherlands; 16 Department of Internal Medicine, University Medical Center Utrecht, Utrecht, Netherlands

**Keywords:** allocation, kidney paired donation, kidney transplantation, living donor, simulation

## Abstract

Kidney exchange programs (KEPs) enhance access to living donor kidney transplantation. Nonetheless, transplant rates in KEP remain low for highly immunized and blood type O patients. In the Netherlands, a novel allocation algorithm is being implemented, allowing ABO-incompatible matching for long waiting patients, next to prioritization and ‘low-level’ HLA-incompatible matching for selected highly immunized patients. We simulated this novel algorithm along with additional scenarios, by using a retrospective, 6-year cohort of Dutch KEP. For each scenario, 30 simulations were repeated with Monte Carlo technique. The novel algorithm increased median KEP transplant rate for incompatible pairs (53% versus 44%, p < 0.001) and for difficult-to-match subgroups. HLA-incompatible matching increased transplant rate for selected highly immunized patients significantly, while participation with multiple donors per recipient did not. In additional simulations, including all non-KEP unspecified donors (n = 150) for local KEP participation increased transplant rate for incompatible pairs up to 64% (p < 0.001). Simulating additional KEP participation by compatible pairs (n = 149), on the condition a KEP match should have fewer HLA mismatches, resulted in 58% being matched in KEP. In conclusion, differential matching algorithms can boost KEP transplant rates, allowing incompatible matching for difficult-to-match subgroups, facilitating participation of unspecified donors, and optimizing the HLA matching of compatible pairs.

## Graphical Abstract

**Figure:**
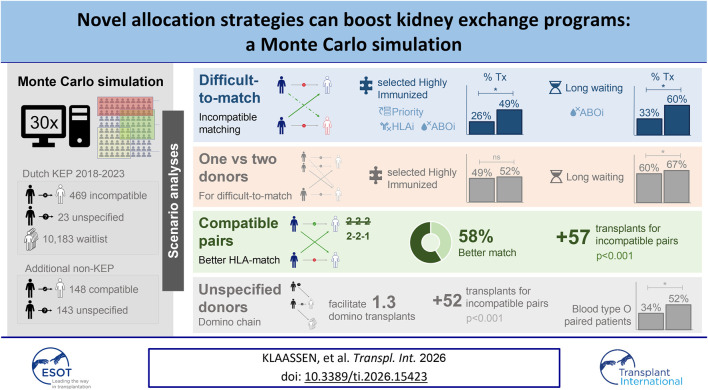

## Introduction

Living donor kidney transplantation (LDKT) is the preferred treatment for end-stage kidney disease, especially when performed pre-emptively [1–4]. For patients with a living donor, blood type (ABO) or Human Leukocyte Antigen (HLA) incompatibility poses barriers in roughly one-third of cases [5]. ABO-incompatible (ABOi) transplantation is common practice in many centers to overcome ABO incompatibility, but it is not feasible in case of high blood type antibody titers and it comes with additional treatment for the recipient [6]. While ABOi transplantation is favorable compared to waiting for a deceased donor transplantation, it is slightly inferior compared to compatible LDKT [7, 8]. HLA-incompatible (HLAi) transplantation is associated with higher graft failure rates despite desensitization protocols [9, 10]. Nonetheless, some degree of HLA incompatibility could be accepted to avoid long-term dialysis with associated morbidity and mortality [11].

Kidney exchange programs (KEPs) can facilitate compatible LDKT by making new donor exchange combinations between incompatible pairs [12, 13]. While single center KEPs can benefit from simplified logistics, multi-center KEPs have proven more effective due to the increased donor pool size [14–18]. The first national KEP was established in the Netherlands over two decades ago [19]. During the years, KEP exchanges have evolved from two-way cycles to four-way cycles and domino chains [20–22]. Unspecified donors and compatible pairs nowadays participate in some KEPs [17, 23–26].

Despite these developments, transplant rates in KEP remain low for difficult-to-match subgroups. Worldwide, highly immunized and blood type O patients accumulate in KEP, leading to inequities in access to kidney transplantation [27–31]. A previous simulation study has shown benefit of allowing ABOi matching for highly immunized patients [24]. Studies from the United States have demonstrated the feasibility of prioritizing matches for difficult-to-match patients by assigning them priority points or weights [14, 32]. Other ways to enable more KEP transplants have been suggested, such as participating with multiple potential donors per recipient in KEP [33], or including compatible pairs who could improve their donor-recipient matching via KEP [25, 34–36].

ABOi matching is already allowed in some KEPs, for example in Spain, the United Kingdom, Scandiatransplant and Australia [20, 27, 37]. In the Netherlands, a new KEP algorithm is being implemented, allowing ABOi matching for long waiting candidates. In addition, selected highly immunized patients are prioritized, by maximizing the number of matches for them first, and are allowed for ABOi or ‘low-level’ HLAi matching [38]. In a single center pilot of this novel algorithm, both the total number of transplants and the number of transplants for difficult-to-match candidates increased when compared to current KEP outcomes [39].

The incremental value of allowing ABOi and HLAi matching, priority allocation, and additional unspecified donor and compatible pair participation in a multi-center KEP has not yet been deciphered. We therefore conducted a simulation study using real-world patient data. The Dutch KEP served as a proxy to test several algorithms and varying numbers of KEP participants. By comparative analysis, we examined how different allocation strategies affect transplant rates across patient groups in KEP.

## Patients and Methods

The MONTEKEP study protocol was approved by the institutional review board of the Erasmus Medical Center (MEC-2023-0518). The need for informed consent was waived.

### Retrospective Data Collection

All Dutch KEP participants from the seven different transplant centers, next to all Dutch patients on the Eurotransplant deceased donor waitlist, from January 2018 to December 2023, were included in the simulation database. We excluded patients with missing data or incomplete waitlist registration. Additionally, we included unspecified donors (UDs) that donated a kidney in their local transplant center outside the national KEP (‘non-KEP UDs’). To simulate additional KEP participation of compatible pairs, we added a sample of non-KEP compatible pairs from one center (Erasmus MC) who had been transplanted with their intended donor, with HLA A-B-DR mismatches ≥4. This sample represents roughly one-fifth of the directed compatible transplants performed nationally with mismatches ≥4.

Waitlist variables such as age, blood type, transplant center, dialysis vintage, HLA A-B-C-DR-DQ (and if available DP) phenotype, and unacceptable HLA antigens were collected. Virtual panel reactive antibodies (vPRA) were calculated from the unacceptable HLA antigens by using the Eurotransplant Reference Laboratory batch calculator^1^. HLA mismatches were calculated on broad level for HLA-A/B and on split level for HLA-DR, in line with the Eurotransplant policy.

### Definitions for Difficult-To-Match Patients

Selected Highly Immunized (sHI) was defined as vPRA≥85%, dialysis vintage ≥2 years, and either ≥2 years participation in the Eurotransplant Acceptable Mismatch (AM) Program or being declined for the AM Program. Long Waiting (LW) was defined as dialysis vintage ≥2 years. Patients were retrospectively labeled as either LW, sHI, HI-other (vPRA≥85% without being LW or sHI), or regular.

### Current and Novel Allocation Algorithms

For the current and novel algorithms, we refer to Table 1. In the novel algorithm, sHI patients are assigned priority and allowed for ‘low-level MFI’ HLAi matching, defined as Luminex SA Werfen Immucor® <8,000 or Thermo Fischer One Lambda® <10,000 and not being a repeated mismatch [40–42]. HLAi matching could be simulated for one center only, as MFI levels of HLA antibodies were only available for patients from one center. ABOi matching is allowed for LW and sHI patients with low IgG anti-A/B titers (≤1:256). We imputed the blood type antibody titers based on the cohort of difficult-to-match patients participating in the pilot KEP in Erasmus MC (Supplementary Table S1) [43]. Blood type A and B patients were assigned to have unacceptably high anti-A/B titers in 3% of cases, compared to 37% among blood type O patients.

**TABLE 1:** Current and novel allocation algorithm of the Dutch kidney exchange program.

Current algorithm	Novel algorithm
1. Maximize the total number of transplants for pairs 2. Blood group identical over blood group compatible 3. Choose matches for patients with lowest match probability (% of HLA- and ABO-compatible donors in that run) 4. Minimize chain length 5. Maximize spread of matches over the centers (for logistical reasons) 6. Choose matches for patients with longest dialysis vintage	1. Maximize the total number of transplants for sHI (compatible → ABOi → HLAi → combined ABOi/HLAi) 2. Maximize the total number of transplants (compatible → ABOi for those allowed) 3. Choose matches for patients with higher vPRA (categorical: 85%-100% → 51%-84% → 6%-50% → 0%-5%) 4. Minimize the length of the longest chain/cycle 5. Choose matches for patients with longer dialysis vintage (categorical: >3 years → 1-3 years → <1 year)

ABOi = ABO-incompatible; HLA, human leukocyte antigen; HLAi = HLA-incompatible; sHI, selected Highly Immunized; vPRA, virtual Panel Reactive Antibodies.

In the novel algorithm, UDs can opt for local KEP participation if they are not willing to travel to another transplant center. Local participation of UDs is limited to matches with pairs from their local transplant center only, while these pairs can still trigger a national domino chain. The last donor kidney of UD-triggered domino chains (end-of-chain kidney) is allocated to the deceased donor waitlist. For this reason, waitlist patients are also assigned LW or sHI status if applicable.

We simulated the following scenarios:

1. Current KEP algorithm.
2. Novel algorithm (=ABOi matching for LW and sHI, and priority for sHI).
3. Novel algorithm + ‘low-level MFI’ HLAi matching for sHI (simulated for one center).
4. Novel algorithm + inclusion of two donors per LW and sHI KEP patient for simultaneous KEP participation.
5. Novel algorithm + inclusion of all UDs in KEP, with either:
  - Local participation (allowing UDs for matching within their local center only).
  - National participation (matching not limited to the local transplant center).
6. Novel algorithm + inclusion of extra compatible pairs with HLA A-B-DR mismatches ≥4. At least one of the following criteria was obliged for matching:
  - Reduction in total number of HLA mismatches on A-B-DR, and ≤1 increase in HLA mismatches on DR locus.
  - Reduction in HLA mismatches on DR locus with no increase in total number of HLA mismatches on A-B-DR.

### Monte Carlo Simulation

Simulations were performed with the Monte Carlo method to predict the most likely effect of an intervention. For a detailed description we refer to the Supplementary Material. For each scenario, 30 simulations were performed. One KEP simulation consisted of 24 KEP match runs, similar to the 24 actual match runs (once every 3 months) in the Dutch KEP during the 6-year study period.

For each simulation, new pairs were created by randomly selecting a donor and patient from the database, after which they were stratified based on incompatibility (ABOi, HLAi, combined ABOi and HLAi) and patient’s status (LW, sHI, HI-other or regular). Stratification percentages and numbers of KEP participants were based on the historical data provided by the Dutch Transplant Foundation (Supplementary Table S1). For the scenario of two donors per difficult-to-match patient (scenario 4), the HLA data of the non-KEP unspecified donors was used to simulate a broadening of the HLA donor pool.

Probabilities of reneging (withdrawal from the program due to medical/non-medical reasons or transplantation outside KEP) and match failure (decline of KEP match due to positive cross-match or medical/non-medical reasons) were taken into account (Supplementary Table S1), based on empirical findings in the Dutch KEP from 2003 to 2011 [24]. Compatible pairs and UDs participated for a maximum of one KEP run in each scenario.

### Outcome Measures

For each scenario, the median transplant rates of 30 simulations were calculated per subgroup, blood type and patient’s status (LW or sHI). Quality of the matches was assessed by reporting the number of ABOi and/or HLAi matches. For the sake of comparison, we compared the outcome data of the Dutch KEP with a base-case simulation of the current Dutch KEP algorithm (scenario 1). The simulated current KEP was the comparator for the simulated novel algorithm (scenario 2). Variants in the novel algorithm and in the number of KEP participants (scenarios 3-6) were compared to the simulated novel algorithm (scenario 2).

### Statistics

Donor and patient characteristics were reported with median and interquartile range (non-normally distributed data). Transplant rates in the real Dutch KEP were compared between the patient subgroups with the z-test. For simulated KEP scenarios, transplant rates were compared with the sign test. Simulated current KEP was compared to real Dutch KEP outcomes with the one sample sign test. P-values <0.05 were considered statistically significant. Data was stored and processed in Microsoft Excel. Statistical calculations were performed with IBM SPSS version 28.0.1 software. Box-and-whisker plots were created with Graphpad Prism 10.1.2. Whiskers depict the minimum and maximum values.

## Results

### Retrospective Cohort

Between 2018–2023, 500 pairs were registered in the national KEP registry, of which 487 were included in this study. Five pairs were excluded for missing data, and eight registrations were re-registrations of similar pairs. Of the 10,487 waitlist patients, 295 were excluded due to incomplete or pending registration. Of the sample of non-KEP compatible pairs, 148 (39%) had HLA mismatches ≥4 and were included in this simulation.

The majority of incompatible KEP patients had blood type O (Table 2). Ratio of blood type O donors versus O patients was 0.4 for incompatible KEP, 2.2 for compatible KEP, and 1.7 for the additional cohort of compatible pairs (Table 3). Of the 166 UDs, the majority (n = 143; 86%) did not participate in KEP and donated in their local center.

**TABLE 2:** Characteristics of patients in the retrospective database.

Characteristic	Paired KEP patients		Waitlist patients	Non-KEP compatible patients
	Incompatible	Compatible		
Number	469	18	10,183	148
Male (%)	232 (50)	10 (56)	6,278 (62)	88 (59%)
Median age (IQR)^a^	54 (45–63)	54 (36–63)	58 (46–66)	59 (49–65)
Blood type (%)	​	​	​	​
A	118 (25)	8 (44)	3,963 (39)	65 (44)
B	54 (12)	4 (22)	1,334 (13)	15 (10)
O	290 (62)	5 (28)	4,469 (44)	65 (44)
AB	7 (1)	1 (6)	417 (4)	3 (2)
On dialysis (%)	275 (59)	13 (72)	5,503 (54)	51 (34)
Median days of patients on dialysis (IQR)^b^	752 (477-1,178)	308 (140-486)	784 (440-1,331)	464 (328-662)
Accepted for AM (%)	65 (14)	0 (0)	286 (3)	0 (0)
Immunized (%)	253 (54)	6 (35)	2,116 (21)	19 (13)
Median % vPRA of immunized (IQR)^b^ ^,^ ^c^	81 (56–99)	37 (23–61)	61 (30–89)	31 (9–56)
Status^b^ (%)				
LW	92 (20)	*NA*	2,627 (26)	NA
sHI	54 (11)	*NA*	325 (3)	NA
HI-other	45 (10)	0 (0)	212 (2)	0 (0)
Regular	278 (59)	18 (100)	7,019 (69)	148 (100)

AM, eurotransplant acceptable mismatch program; HI, highly immunized; IQR, interquartile range; KEP, kidney exchange program; LW, long waiting; sHI, selected Highly Immunized; vPRA, virtual Panel Reactive Antibodies.

^a^ At start of follow-up (1-1-2018) or at registration on the waitlist (if registered after 1-1-2018).

^b^ At end of follow-up (20-12-2023) or at date of transplantation/waitlist removal.

^c^ Calculated with Eurotransplant Reference Laboratory calculator based on unacceptable antigens registered on the Eurotransplant deceased donor waitlist at the end of follow-up (20-12-2023) or at date of transplantation/waitlist removal.

**TABLE 3:** Characteristics of donors in the retrospective database.

Characteristic	Paired KEP donors		KEP UDs	Non-KEP UDs	Non-KEP compatible donors
	Incompatible	Compatible			
Number	469	18	23	143	148
Median age (IQR)^a^	53 (44–61)	51 (40–54)	60 (46–69)	62 (50–68)	55 (48–63)
Blood type (%)					
A	270 (58)	6 (33)	13 (57)	62 (43)	36 (24)
B	76 (16)	1 (6)	2 (9)	14 (10)	2 (1)
O	114 (24)	11 (61)	8 (35)	62 (43)	110 (74)
AB	9 (2)	0 (0)	0 (0)	5 (3)	0 (0)
Ratio blood type O donors to O patients	0.4	2.2	*NA*	*NA*	1.7

IQR, interquartile range; KEP, kidney exchange program; UD, unspecified donor.

^a^ At start of follow-up (1-1-2018) or at registration on the waitlist (if registered after 1-1-2018).

When labelling patients as difficult-to-match, 20% of incompatible KEP patients and 26% of waitlist patients met the criteria for LW status, while this was 11% and 3% for sHI status, respectively (Table 2). sHI KEP pairs more often had blood type O donors than O patients (ratio 1.3), while LW KEP pairs and regular KEP pairs had less blood type O donors than O patients (ratio 0.4 and 0.2, respectively, Supplementary Table S2).

### Reality Versus Simulated Current KEP

In reality, 500 pairs participated between 2018-2023, of which 152 (30%) were transplanted via KEP and 182 (36%) outside national KEP (Supplementary Table S3). Transplant rates in KEP were significantly lower for difficult-to-match subgroups of LW (23%, p = 0.029) and sHI KEP patients (9%, p < 0.001) when compared to regular KEP patients (35%) (Supplementary Table S4). KEP transplant rate for blood type O patients was significantly lower when compared to other blood types (19% versus 47%, p < 0.001).

**FIGURE 1:**
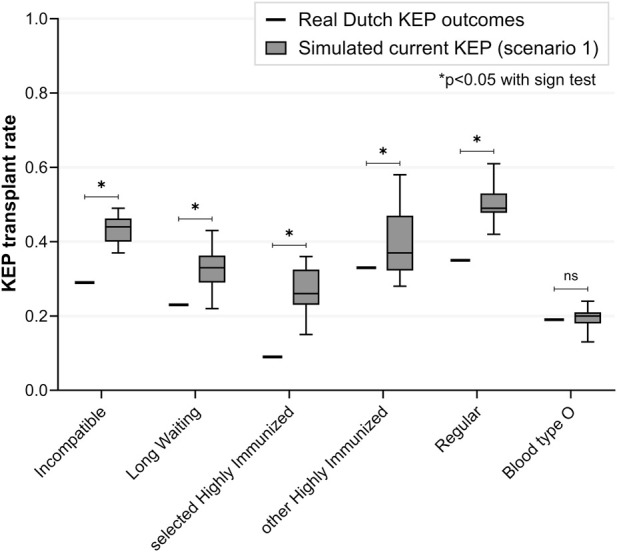
Real versus simulated transplant rates of the current Dutch kidney exchange program *(base case scenario 1)*. Simulated transplant rate for incompatible pairs is significantly different compared to transplant rate in real Dutch KEP. Transplant rate is especially different for selected highly immunized patients, while it is similar for blood type O patients. KEP = Kidney Exchange Program; ns = non-significant.

The simulated current KEP (scenario 1) yielded different transplant rates than real Dutch KEP (Figure 1). Total transplant rate for incompatible pairs was 44% in simulation versus 29% in real Dutch KEP (p < 0.001). Especially, transplant rate was higher in simulation for sHI KEP patients (26% versus 9%, p < 0.001) and blood type A and B patients (Supplementary Table S4). Transplant rate was similar for blood type O KEP patients (20% versus 19%, p = 0.122). All 25 participating UDs were matched in simulation, compared to 11 of 23 in reality.

### Scenario 2: Simulated Novel Algorithm

**FIGURE 2:**
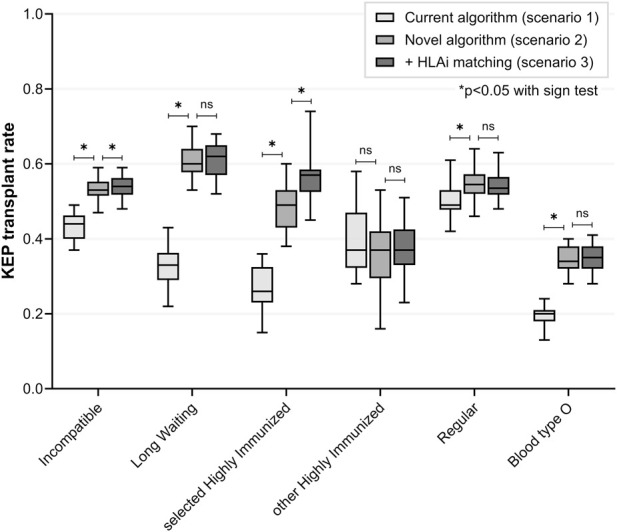
Simulated current versus novel kidney exchange algorithms *(scenarios 1, two and 3).* Priority for selected highly immunized patients and ABO-incompatible matching for both long waiting and selected highly immunized patients significantly increased KEP transplant rate for these difficult-to-match subgroups, without impacting regular patients. HLA-incompatible matching further improved KEP transplant rate for selected highly immunized patients. Transplant rate for blood type O KEP patients was increased with the novel algorithm. HLA = Human Leukocyte Antigen; KEP = Kidney Exchange Program; ns = non-significant.

Compared to simulated current KEP (scenario 1), the simulated novel algorithm (with priority and ABOi matching) resulted in median 50 extra transplants for incompatible pairs (53% versus 44%, p < 0.001) (Figure 2; Table 4). For 98 (67%) of LW and sHI KEP patients, ABOi matching was allowed. ABOi matching and priority resulted in increased transplant rate for difficult-to-match subgroups: 60% versus 33% (p < 0.001) for LW and 49% versus 26% (p < 0.001) for sHI KEP patients (Table 4). Median 50 (17% of total) transplants were ABOi (Table 5). Transplant rate remained similar for HI-other KEP patients (p = 0.541). More regular patients were transplanted with the novel algorithm (55% versus 49%, p < 0.001). Transplant rate for blood type O patients was increased in the novel compared to current algorithm (34% versus 20%, p < 0.001). More end-of-chain kidneys were allocated to sHI waitlist patients (25 versus 10, p < 0.001). Two sHI waitlist patients were matched ABOi.

**TABLE 4:** Number of simulated transplants for each scenario and patient subgroup.

Subgroup	Stratification settings (%)	Median number of transplants (median % of participants)						
		Current KEP	Novel KEP	+ HLAi matching	+ Two donors	+ UDs local	+ UDs national	+ Compatible pairs
		(1)	(2)	(3)	(4)	(5a)	(5b)	(6)
KEP pairs^a^	*499 / 648*	220 (44)	265 (53)	270 (54)	273 (55)	319 (64)	316 (63)	411 (63)
A patients	*NA*	109 (69)	118 (74)	121 (75)	121 (75)	122 (77)	126 (80)	168 (74)
B patients	*NA*	51 (82)	50 (80)	50 (81)	51 (84)	49 (81)	50 (83)	62 (82)
AB patients	*NA*	5 (83)	6 (88)	6 (91)	6 (88)	6 (96)	6 (90)	9 (89)
O patients	*NA*	54 (20)	92 (34)	93 (35)	95 (36)	142 (52)	136 (50)	176 (51)
A donors	*NA*	102 (36)	134 (47)	137 (48)	135 (49)	166 (61)	159 (58)	182 (59)
B donors	*NA*	67 (47)	83 (58)	86 (58)	85 (59)	94 (67)	95 (68)	93 (65)
AB donors	*NA*	5 (31)	6 (38)	6 (43)	8 (44)	11 (50)	12 (52)	6 (40)
O donors	*NA*	46 (78)	46 (82)	48 (83)	47 (79)	52 (81)	55 (83)	129 (70)
Incompatible^a^	*480*	210 (44)	256 (53)	259 (54)	263 (55)	308 (64)	307 (64)	318 (66)
LW	*91 (19)*	30 (33)	55 (60)	56 (62)	61 (67)	62 (68)	62 (68)	62 (68)
sHI	*53 (11)*	14 (26)	26 (49)	30 (57)	28 (52)	27 (50)	28 (53)	27 (51)
HI-other	*43 (9)*	16 (37)	16 (37)	16 (37)	17 (40)	17 (40)	18 (42)	21 (49)
Regular	*293 (61)*	145 (49)	159 (55)	157 (54)	159 (54)	206 (70)	197 (67)	203 (69)
Compatible	*19 / 168*	11 (58)	10 (53)	11 (58)	9 (47)	10 (53)	11 (58)	97 (58)
Unspecified donors	*25 / 175*	25 (100)	25 (100)	25 (100)	25 (100)	175 (100)	175 (100)	25 (100)
Waitlist^a^	*1,440*	25	25	25	25	175	175	25
LW	*792 (55)*	8	0	0	0	42	9	0
sHI	*331 (23)*	10	25	25	25	119	162	25
HI-other	*216 (15)*	5	0	0	0	9	4	0
Regular	*101 (7)*	1	0	0	0	6	1	0
A patients	*NA*	5	12	12	11	88	70	13
B patients	*NA*	17	10	10	10	37	34	10
AB patients	*NA*	3	2	2	2	10	10	1
O patients	*NA*	0	1	1	2	40	61	1

ABOi = ABO-incompatible; HI, highly immunized; HLAi = HLA-incompatible; KEP, kidney exchange program; LW, long waiting; NA, not applicable; sHI, selected Highly Immunized; UD, unspecified donor; vPRA, virtual Panel Reactive Antibodies.

^a^ Median transplant rates of subgroups do not necessarily add up to total transplant rates, as the median of 30 simulations is reported for each subgroup.

**TABLE 5:** Number of incompatible transplants resulting from each simulated scenario.

Subgroup	Median number of incompatible transplants (median % of all transplants)^a^					
	Novel KEP	+ HLAi matching	+ Two donors	+ UDs local	+ UDs national	+ Compatible pairs
	(2)	(3)	(4)	(5a)	(5b)	(6)
ABOi transplants^b^	50 (17)	49 (16)	52 (17)	82 (17)	92 (19)	50 (11)
LW KEP patients	38 (69)	38 (67)	40 (65)	36 (59)	37 (59)	39 (63)
sHI KEP patients	10 (38)	10 (31)	10 (34)	10 (37)	9 (32)	9 (34)
LW waitlist patients	0 (0)	0 (0)	0 (0)	3 (8)	0 (0)	0 (0)
sHI waitlist patients	2 (8)	2 (8)	2 (8)	33 (28)	47 (29)	2 (8)
A KEP patients	5 (4)	5 (4)	5 (4)	5 (4)	4 (3)	5 (3)
B KEP patients	3 (6)	3 (6)	3 (7)	5 (9)	5 (10)	3 (5)
AB KEP patients	0 (0)	0 (0)	0 (0)	0 (0)	0 (0)	0 (0)
O KEP patients	41 (42)	39 (42)	40 (42)	35 (25)	38 (27)	41 (23)
HLAi transplants^b^	NA	3 (1)	NA	NA	NA	NA
sHI KEP patients	NA	3 (9)	NA	NA	NA	NA
sHI waitlist patients	NA	0 (0)	NA	NA	NA	NA
Combined ABOi/HLAi^b^	NA	2 (1)	NA	NA	NA	NA
sHI KEP patients	NA	2 (7)	NA	NA	NA	NA
sHI waitlist patients	NA	0 (0)	NA	NA	NA	NA

ABOi = ABO-incompatible; DDWL, deceased donor wait list; HLAi = HLA-incompatible; KEP, kidney exchange program; LW, long waiting; NA, not applicable; sHI, selected Highly Immunized; UD, unspecified donor.

^a^ Percentages are calculated over the total number of transplants for all patients/for the subgroups of sHI/LW, and blood type.

^b^ Median rates of subgroups do not necessarily add up to total rates, as the median of 30 simulations is reported for each subgroup.

### Scenario 3: Additional HLAi Matching

For 14 (26%) of sHI KEP and 78 (24%) of sHI waitlist patients, MFI levels of HLA antibodies were available. Compared to scenario 2, allowing ‘low-level MFI’ HLAi matching for these patients increased total transplant rate for sHI KEP patients (57% versus 49%, p < 0.001). A median of 3 sHI patients were matched HLAi, and 2 patients both HLAi and ABOi (Table 5). Transplant rates for the other incompatible KEP patients did not change significantly (Figure 2; Table 4).

### Scenario 4: Two Donors Per Difficult-To-Match Patient

**FIGURE 3:**
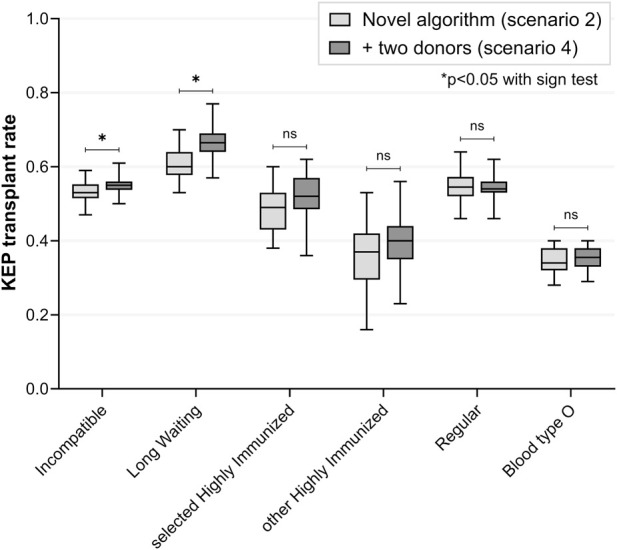
Simultaneous participation by one versus two potential donors per difficult-to-match patient *(scenario 4 versus 2)*. In both scenarios, the novel algorithm with priority and ABO-incompatible matching was simulated. In scenario 4, a second donor was added for each long waiting or selected highly immunized KEP recipient, to participate in KEP with two donors simultaneously. After one of two paired donors was matched, the remaining donor was excluded from the KEP pool. KEP transplant rate for long waiting patients was increased when including two donors versus one, while transplant rate for selected highly immunized patients was not significantly different between the scenarios. KEP = Kidney Exchange Program, ns = non-significant.

Compared to scenario 2, participation of two potential donors per LW and sHI KEP patient increased transplant rate for incompatible KEP patients (55% versus 53%, p = 0.031) (Figure 3; Table 4). Transplant rate for LW KEP patients was slightly increased (67% versus 60%, p < 0.001), but transplant rate was not significantly different for sHI KEP patients (52% versus 49%, p = 0.584). The number of ABOi transplants remained similar (p = 0.345, Table 5).

### Scenario 5: Participation by Extra Unspecified Donors

**FIGURE 4:**
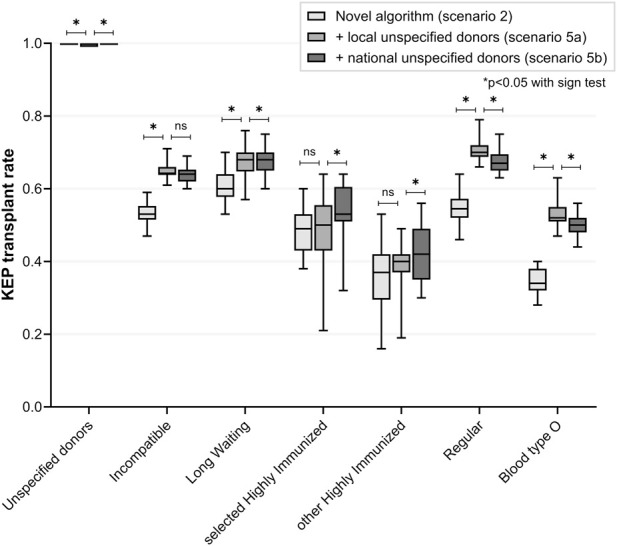
Including all unspecified donors in the novel kidney exchange program *(scenario 5 versus 2)*. In scenario 5a, matching of extra unspecified donors (n = 150) was restricted to a pair from the local center only, after which that pair could trigger a national chain. This local participation by unspecified donors allows them to remain at their evaluating transplant center, eliminating the need to travel to another center for donation (in Dutch KEP there is no organ shipment and the donor travels). In 5b, matching was allowed with pairs from all centers nationally. KEP transplant rate for incompatible pairs was significantly increased in both scenarios with extra unspecified donor participation, especially for blood type O patients and regular patients. National instead of local participation resulted in higher transplant rates for highly immunized subgroups. KEP = Kidney Exchange Program; ns = non-significant.

In scenario 5a, local KEP participation was simulated for 150 additional, non-KEP UDs. All UDs (median 100%) were matched after participating one KEP run (Figure 4; Table 4). Compared to scenario 2, local KEP participation by additional UDs resulted in a median of 52 additional KEP transplants for incompatible pairs via domino chains (transplant rate 64% versus 53%, p < 0.001). Local UD participation facilitated more KEP transplants for blood type O KEP patients (52% versus 34%, p < 0.001). Transplant rate was increased for LW (68% versus 60%, p < 0.001) and regular KEP patients (70% versus 55%, p < 0.001), but remained similar for sHI KEP (p = 1.000) and HI-other KEP patients (p = 0.345). The number of ABOi transplants increased (82 versus 50, p < 0.001), but the proportion of total transplants was similar (p = 0.678). End-of-chain kidneys were mainly allocated to sHI waitlist patients (median 119, 68%) and to blood type A waitlist patients (median 88, 51%). A median of 33 sHI waitlist patients and 3 LW waitlist patients were matched ABOi.

National instead of local UD participation slightly increased transplant rate of highly immunized KEP patients (both sHI and HI-other KEP patients, Figure 4; Table 4). Transplant rate for blood type O KEP patients was reduced (136 versus 142, 50% versus 52%, p < 0.001). However, end-of-chain kidneys were more often allocated to blood type O waitlist patients (61 versus 40, p < 0.001) and sHI waitlist patients (162 versus 119, p < 0.001) with national UD participation. The proportion of ABOi transplants increased when compared to the local UD scenario (19% versus 17%, p < 0.001, Table 5).

### Scenario 6: Participation by Extra Compatible Pairs

**FIGURE 5:**
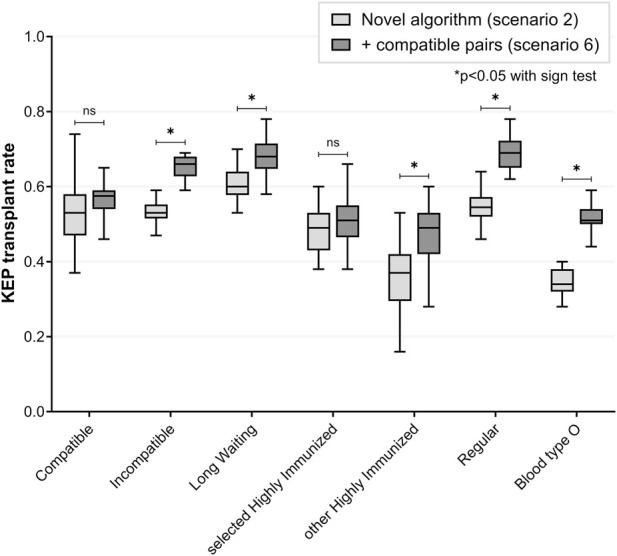
Additional participation of compatible pairs in the novel kidney exchange program *(scenario 6 versus 2).* A sample of compatible pairs (n = 149), representing roughly one-fifth of national directed compatible transplants with HLA A-B-DR mismatches ≥4, was included for KEP participation. For these compatible pairs, a KEP match was restricted to fewer total HLA A-B-DR or fewer HLA-DR mismatches, and participation was limited to a single match run. More than half of compatible pairs could be matched with a KEP donor on these conditions. Compatible pair participation increased transplant rate for incompatible pairs and for difficult-to-match subgroups of long waiting and blood type O patients. KEP transplant rate for selected highly immunized patients remained similar. HLA = Human Leukocyte Antigen; KEP = Kidney Exchange Program; ns = non-significant.

Additional KEP participation was simulated for 149 compatible pairs, which represented roughly one-fifth of the national compatible directed transplants with HLA mismatches ≥4. This resulted in 58% of compatible pairs being matched with a donor with fewer total HLA A-B-DR or fewer HLA DR mismatches, after participating one KEP run (Figure 5; Table 4). Compatible KEP participation increased transplant rate for incompatible pairs (median +57, 66% versus 53%, p < 0.001), and for the subgroups of LW KEP (68% versus 60%, p < 0.001), HI-other KEP (49% versus 37%, p < 0.001) and regular KEP patients (69% versus 55%, p < 0.001). Transplant rate for sHI KEP patients was similar (51% versus 49%, p = 0.078). Transplant rate for blood type O KEP patients increased (51% versus 34%, p < 0.001): explained by more transplants for both compatible and incompatible blood type O KEP patients (median 146 versus 91 for incompatible, p < 0.001). Number of ABOi transplants remained similar compared to scenario two (p = 0.850, Table 5).

## Discussion

Kidney exchange programs facilitate living donor kidney transplantation, thereby reducing the waitlist and avoiding long-term dialysis. Additionally, KEPs reduce incompatibilities between donor and recipient, mitigating the need for augmented immunosuppression and ultimately improving long-term graft survival [4, 13, 27–30, 39]. We simulated various KEP scenarios to optimize allocation strategies and solve common KEP problems, such as the congestion of highly immunized and blood type O patients.

In our study, ABOi matching for difficult-to-match patients resulted in a higher transplant rate for this group, while concomitantly increasing the transplant rate for regular patients. Scandiatransplant, which allows ABOi matching by default, has reported a transplant rate of 40% after 4 years [27]. This is in line with our simulation findings, as more HLAi pairs participate in Scandiatransplant and KEP volume is lower compared to the Netherlands. The United Kingdom Living Kidney Sharing Scheme also allows ABOi matching, but reports low rates of KEP ABOi transplants (seven over the past 4 years) [44]. Interestingly, Manook et al. described a trend in the United Kingdom to perform less direct, incompatible transplants, with a shift to more and longer KEP participation of incompatible pairs [45].

In line with previous reports, prioritizing highly immunized patients did not decrease the transplant rate for regular patients [32, 38]. Ashlagi et al. has simulated that, despite a priority point scale, the KEP transplant rate of patients with PRA >98% still lags behind those of other immunized patients [14]. This is a well-known phenomenon in deceased donation programs as well, with a subgroup of very immunized patients not being transplanted despite priority [46, 47]. As priority alone would not suffice for this group, HLA-incompatible transplantation within KEP for lower-risk HLA antibodies might be the best alternative to dialysis or higher-risk incompatible transplantation outside KEP [47–49]. Our simulations demonstrate that HLA-incompatible matching, based on MFI cut-off values predicting a low risk of a positive crossmatch, would lead to more matching opportunities for this difficult-to-match group.

Compatible pairs can participate in KEP for altruistic reasons or to improve their immunological matching via KEP, by reducing the number of HLA mismatches or by avoiding repeated mismatches from previous transplants or pregnancies [50]. This can potentially improve long-term graft survival, reduce the risk of immunization, and prevent future re-transplantation [4]. In our simulations, an improved HLA match was found for more than half of compatible pairs, after participating one KEP run. In line with three other simulation studies [26, 34, 35], KEP participation by compatible pairs increased the transplant rate for incompatible pairs. Of note, Gentry et al. simulated two-way exchanges only [26], while the two other studies simulated a single KEP match run only. [34, 35]. In our simulation, participation of compatible pairs did not result in extra transplants for highly immunized patients, although the immunized patients were prioritized. This unexpected finding, possibly related to changes in the KEP pool composition on the long term, demonstrates the importance of simulating multiple KEP runs over extended time periods. As we defined HLA matching on the antigen level, future studies on compatible KEP participation could test epitope matching for better immunological risk assessment [34, 51, 52].

In deceased donation, it has been demonstrated that internationally merged pools could enhance matching options for highly immunized patients [53]. In KEPs, it is known that a larger pool size allows for more and ‘better’ matches [18]. International cooperation between KEPs has been shown feasible and beneficial for KEP transplant rate [16, 27, 54], though logistical challenges must be overcome [15]. In this study, we tested enlarging the KEP donor pool via the inclusion of two potential donors per (difficult-to-match) recipient. We found no statistically significant difference for highly immunized patients, and only a modest benefit for long waiting patients. Although evaluation of multiple potential donors might increase costs and burden for donor candidates, the benefit may be more evident when including more additional donors or including donors with more diverse HLA genotypes.

Domino paired donation by unspecified donors has already been proven effective [23, 55–57]. Though logistically challenging, countries with low unspecified donation could consider deceased-donor initiated chains, that have a similar boosting effect [58–61]. For KEPs using donor travel as modality (such as in the Netherlands), local KEP participation of unspecified donors or compatible pairs might be a solution to increase KEP participation. Nonetheless, national instead of local participation will result in higher transplant rates for difficult-to-match subgroups. Clinicians should carefully counsel unspecified donors on local versus national KEP participation.

A persistent problem in KEPs is the shortage of blood type O donors [22, 31]. ABOi matching and KEP participation by unspecified donors or compatible pairs can reduce the blood type imbalance. Importantly, when unspecified donors with blood type O participate in KEP, the end-of-chain kidneys will mostly be of blood type A/B, resulting in less blood type O waitlist patients being matched via KEP chains. However, allowing ABOi matching for end-of-chain kidneys will reduce this disadvantage: in our simulations with ABOi matching and national unspecified donor participation, in which a median of 75 unspecified donors with blood type O were included, end-of-chain kidneys were matched (ABO-incompatible) to blood type O waitlist patients in median 61 cases. KEPs could consider prioritizing the matching of end-of-chain kidneys to blood type O waitlist patients, via an ABOi transplant, to further reduce blood type inequities on the waitlist. Of note, ABOi matching will not benefit all blood type O patients, as one-third had titers above threshold for ABOi transplantation in our pilot cohort [43].

We compared our base-case simulation of the current KEP to real Dutch KEP outcomes. There were significant differences: more patients, especially highly immunized patients and blood type A and B patients, were transplanted in simulation. This might be caused by the different blood type distribution, as we did not stratify for blood types of simulated donor-recipient pairs; more blood type B donors were included compared to real Dutch KEP, and less blood type O donors. Additionally, renege or match failure rates might have been higher in reality: renege rate was set to 2% per month based on a historical estimate, while transplantation outside KEP with another living or deceased donor occurred in one-third of KEP pairs during the study period. Especially for unspecified donors, many were not transplanted in real Dutch KEP in this specific period, probably related to the COVID pandemic, while in simulation there was no reneging of unspecified donors. Simulated transplant rates should therefore be interpreted with caution and relative to the base-case scenario.

In summary, we demonstrated the potential of adaptations in a national KEP and revealed the impact of these changes on different patient groups, by using a simulation model with real KEP data. This allows clinicians and policymakers to reflect on the challenges in their respective KEPs and to consider changes to the allocation strategy accordingly. Nevertheless, our study remains a simulation with practical simplifications and assumptions. Importantly, waiting time of ABOi pairs in KEP was not restricted in simulation, while in reality these pairs proceed with directed ABOi transplantation after participating unsuccessfully in one or two KEP runs. Next, our simulation output lacked parameters for comparing the HLA matching between the various scenarios. Additionally, our algorithm did not incorporate a center balance system, which ensures each center receives as many end-of-chain kidneys as the number of unspecified donors they contributed. Due to the retrospective nature of the data, our model did not account for waitlist dynamics, such as changes in dialysis vintage and vPRA over time. For HLAi matching, we simplified the delisting of antibodies by retrospectively applying a strict MFI cut-off, while a personalized approach is recommended [62].

In conclusion, a well-functioning KEP facilitates living donor kidney transplants, reduces incompatibilities and improves HLA matching. Differential matching constraints for difficult-to-match subgroups boost KEP transplant rate and mitigate current inequities. KEP participation by unspecified donors is pivotal and should be facilitated. Mismatched compatible pairs should be counseled to optimize their HLA matching via KEP. Novel allocation strategies should be tested in simulation to predict the effects and evaluate consequences for subgroups of participants.

## Data Availability

Original datasets are available upon request to the corresponding authors.

## Footnotes

1
https://www.etrl.org/FreqBatch.aspx
