# Supplementary material for: Novel Allocation Strategies Can Boost Kidney Exchange Programs: A Monte Carlo Simulation
Source: Transpl Int. 2026 Mar 4;39:15423. doi: 10.3389/ti.2026.15423 (PMC12995821; doi:10.3389/ti.2026.15423)
Supplement: Supplementary file 1 [file DataSheet1.pdf]

SUPPLEMENTARY MATERIAL

Supplementary Table S1: Simulation settings

Simulation settings	
Renege rate*	2% per month for each pair and waitlist patient
Match failure rate#	12.5% + probability dependent on vPRA (ranging from 19-58%)
Maximum cycle / chain length	4
Maximum age difference for compatible pairs	30 years
Maximum HLAi and/or ABOi transplants per match run	10
Default arrival rates (derived from the Dutch KEP between 2018-2023)	
Pairs arrival per 3 months	20
Patient's status stratification:	19% LW, 11% sHI, 9% HI-other, 61% regular
Incompatibility with intended donor stratification:	51% HLAi, 37% ABOi, 12% combined ABOi & HLAi
Ratio of compatible versus incompatible pairs	0.04 *(and up to 0.35 in additional scenarios)*
Waitlist patients arrival per 3 months	60
Patient's status stratification:	55% LW, 23% sHI, 15% HI-other, 7% regular
Altruistic donors arrival per 3 months	1 *(and up to 7 in additional scenarios)*

Blood type antibody titer imputation

Blood type	*Patients with IgG A/B titers >1:256 in pilot cohort#*	*Simulation settings for unacceptable A/B titers*
A	0/24 (1 with titer on threshold)	3% (anti-B)
B	1/36 (3%)	3% (anti-A)
AB	Titers not measured	0%
O	18/48 (13 anti-A, 3 anti-B, 2 both anti-A/B)	37% (72% anti-A, 17% anti-B, 11% both anti-A/B)

*Renege rate represents medical problems with the donor/recipient, being transplanted via other programs or personal decisions of donor/recipient. Match failure rate represents match failure due to positive crossmatch, medical problems with the donor/recipient, or decline of the match by the participating centers. Rates are based on empirical findings in the Dutch KEP from 2003-2011, as described in: *Glorie KM, de Klerk M, Wagelmans AP, et al. Coordinating unspecified living kidney donation and transplantation across the blood-type barrier in kidney exchange. Transplantation. 2013; 96: 814-20.*
#Published in conference abstract: *Klaassen MF, de Klerk M, Kal-van Gestel JA, et al. Blood group antibody titers in long-waiting candidates for ABO-incompatible kidney exchange program transplantation: a retrospective cohort study. Dutch Transplant Society 'Bootcongres' 2024, 35th Annual Meeting. Dutch Transplant Foundation, Utrecht, The Netherlands, 2024. Available on: https://www.transplantatievereniging.nl/wp-content/uploads/2024/03/Programma-Bootcongres-2024-def-2.pdf*

Detailed description of simulation model:

Labeling of difficult-to-match patients: Patients were assigned LW or sHI status based on the unacceptable antigens and dialysis vintage as registered on the waitlist at the end of the study period or at the time of transplantation/removal from the waitlist. Thus, all patients that ever became LW or sHI during the study period were assigned priority and/or allowed for HLAi/ABOi matching at start of inclusion in the simulation. For this study, we considered all patients for which AM participation was not requested after ≥2 years dialysis as rejected for AM. For patients with AM participation, we used the criterium of dialysis vintage ≥4 years for sHI status, as duration of AM participation was not available and AM participation is allowed with dialysis vintage ≥2 years.

Stratification: Participants were randomly extracted from the retrospective database by sampling with replacement, meaning participants could be extracted more than once in each simulation. At the start of each simulation, a random inclusion date was assigned to each participant or pair. Dialysis vintage was recalculated with dialysis start date or set to zero if the assigned inclusion date was before dialysis start date. Before the initial match run, an extra arrival round was performed to construct a pool with comparable size to the real KEP pool in January 2018. Due to computational limitations, only a limited sample of waitlist patients could be included. We therefore increased the stratification percentage for LW and sHI waitlist patients to reach comparable, absolute numbers as in reality. When simulating additional participation by UDs or compatible pairs, we rounded the stratification number (compared to reality) as we had to set an integer number and ratio of participants included in each match run, respectively.

Including two donors per difficult-to-match recipient: For simulating the second donor per recipient in scenario 4, the data of the non-KEP UDs was added to the simulation database to broaden the HLA diversity of the donor pool. Blood type distribution of this additional, second-donor population was corrected to be similar to the LW and sHI KEP donor population. The additional donor was randomly assigned with stratification for incompatibility with the intended recipient. Whenever a match was found for one of the two participating donors, the remaining donor was excluded from the simulation.

Supplementary Table S2: Characteristics of the different patient subgroups in the Dutch kidney exchange program.

	Incompatible KEP patients				Deceased donor waitlist patients			
	LW	*sHI*	*HI-other*	*regular*	*LW*	*sHI*	*HI-other*	*regular*
Number	92	54	45	278	2,627	325	212	7,019
Male (%)	47 (51)	20 (37)	12 (27)	153 (55)	1,692 (64)	158 (49)	77 (36)	4,351 (62)
Median patient age (IQR)*	53 (44-60)	48 (37-60)	52 (48-62)	56 (46-64)	56 (47-64)	51 (40-61)	55 (43-64)	59 (46-67)
Median donor age (IQR)*	53 (43-59)	50 (40-57)	56 (48-63)	54 (45-61)	*NA*	*NA*	*NA*	*NA*
ABO of patient (%)								
A	13 (14)	26 (48)	20 (44)	59 (21)	759 (29)	102 (31)	101 (48)	3,001 (43)
B	10 (11)	8 (15)	9 (20)	27 (10)	455 (17)	52 (16)	24 (11)	803 (11)
O	67 (73)	19 (35)	16 (36)	188 (68)	1,311 (50)	157 (48)	80 (38)	2,921 (42)
AB	2 (2)	1 (2)	0 (0)	4 (1)	102 (4)	14 (4)	7 (3)	294 (4)
ABO of donor (%)								
A	51 (55)	20 (37)	18 (40)	181 (65)	*NA*	*NA*	*NA*	*NA*
B	14 (15)	8 (15)	3 (7)	51 (18)	*NA*	*NA*	*NA*	*NA*
O	25 (27)	25 (46)	23 (51)	41 (15)	*NA*	*NA*	*NA*	*NA*
AB	2 (2)	1 (2)	1 (2)	5 (2)	*NA*	*NA*	*NA*	*NA*
Ratio type blood type O donors to O patients	0.4	1.3	1.4	0.2	*NA*	*NA*	*NA*	*NA*
On dialysis (%)	92 (100)	54 (100)	24 (53)	105 (38)	2,627 (100)	325 (100)	114 (54)	2,437 (35)
median days of patients on dialysis (IQR)#	988 (856-1234)	1,812 (1293-2391)	494 (369-593)	449 (237-610)	1,230 (952-1664)	1,930 (1415-2946)	508 (296-610)	408 (252-574)
In AM program (%)	19 (21)	31 (57)	15 (33)	0 (0)	82 (3)	140 (43)	58 (27)	6 (0.1)
Immunized (%)	49 (53)	54 (100)	45 (100)	105 (38)	600 (23)	325 (100)	212 (100)	979 (14)
median % vPRA of immunized (IQR)#, §	77 (60-99)	99 (97-100)	97 (90-99)	55 (25-68)	56 (29-74)	98 (93-100)	95 (90-99)	42 (23-63)
Incompatibility (%)								
ABO-incompatible	46 (50)	1 (2)	2 (4)	192 (69)	*NA*	*NA*	*NA*	*NA*
HLA-incompatible	34 (37)	39 (72)	35 (78)	66 (24)	*NA*	*NA*	*NA*	*NA*
Combined	12 (13)	14 (26)	8 (18)	20 (7)	*NA*	*NA*	*NA*	*NA*

ABO = ABO blood type system; AM = Acceptable Mismatch; HI = Highly Immunized; HLA = Human Leukocyte Antigen; IQR = Interquartile Range; KEP = Kidney Exchange Program; LW = Long Waiting; NA = Not Applicable; vPRA = virtual Panel Reactive Antibodies.

*At start of follow-up (1-1-2018) or at registration on the waitlist (when registered after 1-1-2018).

#At end of follow-up (20-12-2023) or at date of transplantation/waitlist removal.

§Calculated with ETRL calculator based on unacceptable antigens registered on the Eurotransplant deceased donor waitlist at the end of follow-up (20-12-2023) or at date of transplantation/waitlist removal.

Supplementary Table S3: Number of participants transplanted within and outside the Dutch kidney exchange program between 2018-2023

Outcome	*Number of pairs*
Transplanted in national KEP (%)	152 (30)
Transplanted outside national KEP (%)	182 (36)
with intended living donor kidney (incompatible)	56 (31)
with other living donor kidney (other directed donor / local exchange)	75 (41)
with deceased donor kidney	51 (28)
Removed from KEP list during study period (%)	92 (18)
Still in KEP at the end of the study period (%)	74 (15)
Total	*500*

KEP = Kidney Exchange Program.

Supplementary Table S4: Number of transplants in real versus simulated current kidney exchange program.

Subgroup	Number of KEP transplants (median % of participants)		
	Real Dutch KEP (between 2018-2023)	*Simulated current KEP (median of 30 runs)*	*P-value**
KEP pairs	152 (30)#	220 (44)	<0.001
Incompatible pairs	141 (29)#	210 (44)	<0.001
ABO-incompatible pairs	60 (24)#	81 (33)	<0.001
HLA-incompatible pairs	70 (40)#	102 (58)	<0.001
Combined ABOi/HLAi pairs	11 (19)#	24 (41)	<0.001
LW KEP patients	22 (23)$	30 (33)	<0.001
sHI KEP patients	5 (9)$	14 (26)	<0.001
HI-other KEP patients	15 (33)$	16 (37)	0.043
Regular KEP patients	99 (35)$	145 (49)	<0.001
Compatible pairs	11 (52)#	11 (58)	0.362
Blood type A KEP patients	63 (48)#	109 (69)	<0.001
Blood type B KEP patients	27 (47)#	51 (82)	<0.001
Blood type AB KEP patients	3 (38)#	5 (83)	<0.001
Blood type O KEP patients	59 (19)#	54 (20)	0.122
Unspecified donors	11 (48)#	25 (100)	<0.001
Deceased donor waitlist patients	11#	25	<0.001

HLA = Human Leukocyte Antigen; KEP = Kidney Exchange Program; LW = Long Waiting; sHI = selected Highly Immunized; vPRA = virtual Panel Reactive Antibodies.
*Calculated on transplant rates (% of participants transplanted) with one sample sign test.
#Numbers were collected from the KEP data dashboard of the Dutch Transplant Foundation and can differ from the number of pairs included in the simulation database, as some pairs participated more than once in KEP.
$Calculated over 494 of 500 pair registrations, as 5 pairs with 6 KEP registrations had missing data and retrospective sHI/LW labeling was not possible for them.
